# Emergency management of ureteral stones: Recent advances

**DOI:** 10.4103/0970-1591.44248

**Published:** 2008

**Authors:** Luis Osorio, Estêvão Lima, Riccardo Autorino, Filinto Marcelo

**Affiliations:** Department of Urology, Santo Antonio General Hospital, Oporto, Portugal; 1 Urology Clinic, Second University of Naples, Naples, Italy

**Keywords:** Emergency, management, ureteric colic, ureteral stones

## Abstract

Most ureteral stones can be observed with reasonable expectation of uneventful stone passage. When an active ureteral stone treatment is warranted, the best procedure to choose is dependent on several factors, besides stone size and location, including operators’ experience, patients’ preference, available equipment and related costs. Placement of double-J stent or nephrostomy tube represents the classical procedures performed in a renal colic due to acute ureteral obstruction when the conservative drug therapy does not resolve the symptoms. These maneuvers are usually followed by ureteroscopy or extracorporeal shockwave lithotripsy, which currently represent the mainstay of treatment for ureteral stones. In this review paper a literature search was performed to identify reports dealing with emergency management of renal colic due to ureteral stones. The main aspects related to this debated issue are analyzed and the advantages and disadvantages of each treatment option are carefully discussed.

## INTRODUCTION

Acute renal colic is a common complaint observed in the emergency room. It is usually described as an acute flank pain radiating to the groin and it is often caused by ureteral stones.[1]

The clinical diagnosis should be supported by an appropriate imaging procedure. During recent years, unenhanced helical computed tomography has been introduced as a quick and contrast-free alternative to urography. An alternative and commonly applied method for evaluating patients with acute flank pain is a plain film of kidneys, ureter and bladder combined with ultrasonography.[2]

The first step in the treatment for acute renal colic caused by obstructing ureteral stones is medical relief of symptoms. When a drug therapy does not resolve the symptoms, the placement of a ureteral catheter or a nephrostomy tube has routinely represented the next step.[3] These easy maneuvers can offer a prompt relief from pain for the patient and they are usually followed by ureteroscopy (URS) or extracorporeal shockwave lithotripsy (ESWL), which currently represents the mainstay of treatment for symptomatic ureteral stones.[4]

In the last five years, these two treatment modalities have gained growing attention also in the emergency setting, applied rapidly after the onset of renal colic. Being able to result in both stone disintegration and relief from acute obstruction they represent an attractive option.[5]

The aim of the current report is to critically review the evidence on the emergency management of symptomatic ureteral stones.

## MATERIALS AND METHODS

The present study is based on a structured literature review. A MEDLINE search was performed for publications in the English language using the key words “ureteral stones”, “renal colic”, “emergency management”, “ureteroscopy”, “extracorporeal shockwave lithotripsy”. Inclusion criteria were established before the search was initiated in order to select only relevant full-length papers that met the criteria of the analysis. Therefore, only studies addressing the management in an emergency setting of symptomatic ureteral stones were included and reviewed in detail. Furthermore, papers identified from the reference lists of selected papers were also considered.

## CONSERVATIVE MANAGEMENT

Most ureteral stones can be observed with a reasonable expectation of uneventful stone passage and this strategy is generally less costly and less invasive than any other option, if successful.[6] Ureteral stones with a diameter less than 5 mm will pass in up to 68% of cases; however, for stones with a greater diameter the overall chances of spontaneous passage are lower.[4]

Overall, stone size and position, degree of impaction and of obstruction at the initial presentation are factors influencing the likelihood of and the time to spontaneous passage.[6] It has been recommended that stone passage should not exceed four to six weeks due to the risk of renal damage.[7] Conservative management is not appropriate in patients with risk factors for urosepsis, such as prolonged obstruction, persisting pain or associated infection. Moreover, there is an absolute indication for actively removing urinary stones is some sub-categories of patients, such as pilots or sailors.

An observational approach based only on the pharmacological control of pain, involving the administration of several agents by various routes, has been claimed since pain relief still remains as the most urgent step in patients with an acute stone episode.[8] Increasing fluid flow through the affected kidney may expedite stone passage even if interventions aiming to do so, such as intravenous high volumes or oral fluids and diuretics, have a controversial effect.[9]

Oral diclofenac in the prophylaxis of recurrent renal colic was evaluated in a double-blind placebo-controlled prospective study by Laerum *et al.* They demonstrated this treatment option to be effective in reducing colic and hospital admissions, even if stone passage rate was not affected.[10] Hydromorphone and other opiates without simultaneous administration of atropine should be avoided because of the increased risk of vomiting.[8]

Diclofenac belongs to nonsteroidal anti-inflammatory drugs (NSAIDs). These drugs have also been shown to interfere with the autoregulatory response to obstruction by decreasing renal blood flow. Although the renal function can be affected in patients with an already reduced function, this is not the case for normally functioning kidneys.[11]

Tramadol is more potent than previous oral preparations, with fewer opioid-type side-effects and less potential for dependence.[12] Ketorolac given intramuscularly is as effective as tramadol with an earlier analgesic effect.[13]

## MEDICAL EXPULSIVE THERAPY

Medical expulsive therapy (MET) has recently emerged as an appealing option for the initial management of ureteral stones.[14]

Several pharmacological approaches have been proposed in recent years aiming to act on possible causes of stone retention.[7] Both α-antagonists and calcium channel blockers have been shown to inhibit the contraction of ureteral muscle responsible for ureteral spasms while allowing antegrade stone progression.[15,16]

Even if the literature relating to the optimal conservative regime is sparse, some randomized studies have been reported assessing different drug combinations with encouraging outcomes in terms of expulsion rate, time to expulsion and pain control[17] [Table 1].

**Table 1:** Medical expulsive therapy in the management of symptomatic ureteral stones: Data from the literature

Reference	Regimen	Mean stone size, mm	Observation time, weeks	Expulsion rate (%)	Mean expulsion (days) time
α-antagonists					
21	Tamsulosin	5.4	4	24/28 (86)	7.9
	vs. control	5.4		12/28 (43)	12
22	Tamsulosin	7.2	4	68/70 (97)	3
	vs. control	6.2		45/70 (64)	5
24	Tamsulosin	-	1	41/51 (80)	-
	vs. control	-		32/53 (60)	-
25	Tamsulosin	6.7	4	30 /30 (100)	2.7
	vs. control	5.8		21/30 (70)	4.6
26	Tamsulosin	6.5	4	28/32 (88)	4.8
	vs. control	5.7		19/32 (59)	7.4
27	Tamsulosin	6	4	23/29 (79)	6.3
	Terazosin	6		22/28 (79)	5.8
	Doxazosin	5.9		22/29 (76)	5.9
	vs. control	6.1		15/28 (54)	10.5
28	Tamsulosin	6.9	2	45/50 (90)	4.4
	vs. control	6.4		27/46 (59)	7.5
29	Terazosin	6.9	4	29/32 (91)	3.2
	vs. control	6.6		20/32 (63)	5.9
30	Tamsulosin	5.9	7	51/66 (77)	-
	vs. control	5.7		23/48 (48)	-
31	Tamsulosin	-	4	40/45 (88.9)	7.3
	vs. control	-		23/45 (51.1)	12.5
Calcium channel blockers					
7	Nifedipine	3.9	7	31/35 (89)	12.6
	vs. control	3.9		19/35 (54)	11.2
18	Nifedipine	6.7	7	34/43 (79)	11.2
	vs. control	6.8		24/43 (56)	16.4
19	Nifedipine	5.8	4	38/48 (79)	7
	vs. control	5.5		17/48 (35)	20
20	Nifedipine	12	3	15/25 (60)	6
	vs. control	12.8		12/25 (48)	10
21	Nifeipine	4.7	4	24/30 (80)	9.3
	vs. control	5.4		12/28 (43)	12
22	Nifeipine	6.2	4	54/70 (77)	5
	vs. control	6,2		45/70 (64)	5

Nifedipine: This is a calcium channel blocker commonly used in the treatment of hypertension and angina. It acts as a suppressing mechanism of the fast component of ureteral contraction leaving the peristaltic rhythm unchanged. Its use in medical therapy for distal ureteral lithiasis has been tested in various studies, which have demonstrated its excellent efficacy for inducing stone expulsion and relieving pain, although the lack of validation by multicenter trials has not allowed it to diffuse the proposed treatment regimens.[7,18–22] A pooled data analysis including 686 patients, mostly with distal ureteral stones > 5 mm, suggested a benefit in terms of stone explusion and time to stone expulsion when nifedipine is combined with standard therapy. Overall, adverse effects were observed in 15.2% of patients in these trials.[17]

Tamsulosin: The addition of α-antagonists to routine analgesia has been proposed to facilitate stone passage by inhibiting basal tone, peristaltic frequency and ureteral contractions through their action on the α-1 adrenergic receptors in ureteral smooth muscle.[23] Pooled data form 16 clinical trials including 1235 patients with distal ureteral stones between 3 and 18 mm suggest a benefit in stone expulsion. [24–33] The most commonly used agent was tamsulosin 0.4 mg taken daily for one month. However, in several trials terazosin 5-10 mg daily or doxazosin 4 mg daily were used with similar efficacy. Therefore the benefit is probably a class effect rather than an effect specific to tamsulosin.[17] Moreover, a two- to six-day average improvement in time to stone expulsion was observed in patients receiving an α-antagonist.^17^] The mean time to stone explusion in these patients was less than 14 days, with an overall adverse effects rate of 4%.[17] Thus, according to the available evidence, an adrenergic α-antagonist is an effective adjunct to the standard analgesic therapy in the outpatient pharmacological treatment of uncomplicated distal ureteral stones.[32] It would be within the standard of care to add a short course (two to four weeks) of tamsulosin to analgesic therapy for patients discharged from the emergency department with appropriate urologic follow-up.

## ACTIVE STONE REMOVAL

Active stone removal should be considered for stones with a diameter ≥ 7 mm and when adequate pain relief cannot be achieved, stone obstruction is associated with infection, there is a risk of urosepsis in single kidneys with obstruction or in cases of bilateral obstruction.[3]

When an active ureteral stone treatment is warranted, the best procedure to choose is dependent on several factors, besides stone size and location, including operators’ experience, patient preference, available equipment and related costs.[4]

The standard first-line approach in the management of symptomatic ureteral stone is relief of obstruction by insertion of a nephrostomy tube or a double-J stent and fragmentation of the stone later. Insertion of a nephrostomy tube under local anesthesia is relatively less invasive and it is considered to be better if there is evidence of sepsis at the time of presentation. Nevertheless, its potential disadvantages are leakage, dislodgement of the tube and the need to manage the stoma.[33] Insertion of a double-J stent, apart from the complications such as ureteral perforations and failure to pass the stent in some cases, may increase the risk of urosepsis. Furthermore, the presence of a stent results in a reduction of the shock wave energy reaching the stone, and causes ureteral constriction and edema of the wall, both of which may reduce the chance of successful fragmentation or the passage of fragments after SWL.[34]

Emergency SWL: Shockwave lithotripsy is the most widely used method for managing renal and ureteral stones. However, treatment success rates depend on stone composition, size, and location, as well as instrument type and shock frequency. Since the introduction of SWL for the removal of stones, this procedure has been optimized, and new instruments have been developed to increase utility to urologists and to improve tolerability for the patient.

Shockwave lithotripsy as first-line therapeutic option, applied rapidly after the onset of renal colic, has deserved very limited attention so far. Since the pioneering paper by Doublet et al.,[35] a few papers have been reported in the last years on the effective use of ESWL in emergency conditions, all with encouraging results even if mainly for proximal ureteral stones[36–40] [Table 2]. Seitz and colleagues showed that a gradual increase of the time after a first colic episode until ESWL treatment significantly correlated with delayed stone clearance. Subsequently the same group further highlighted the absence of significant impaction in proximal ureteral stones when treated within 24 h.

**Table 2:** Active emergency treatment in the management of symptomatic ureteral stones: Data from the literature

Author [ref.]	Emergency procedure	N° pts	N° proximal/distal	Mean stone size	SFR (%)
Joshi *et al.*[33]	SWL	16	9/7	8.2 (6.5-10.2)	81
Tligui *et al.*[36]	SWL	200	98/102	7 (3-20)	82
Tombal *et al.*[37]	SWL	50	29/21	6.4 (5.7-6.9)	74
Kravchick *et al.*[38]	SWL	53	53/0	7.1 (5-13)	72
Seitz *et al.*[39]	SWL	91	91/0	7.9 (5.6-10.2)	76.9
Seitz *et al.*[40]	SWL	82	82/0	7.8 (4.6-11)	80.5
Osorio *et al.*[42]	URS	144	14/130	9.1 (5-20)	92.4

SWL – Extracorporeal shockwave lithotripsy; URS – Ureteroscopy; SFR – Stone free rate

The main drawback in these reports remains the need for auxiliary procedures which can be explained by the use of new generation lithotripters.[4

Emergency Ureteroscopy: Ureteroscopy represents a safe and minimally invasive procedure in the management of ureteral stones. Advancements in technology have made it a safe and highly successful procedure, reducing its complication rates.[41] Similar to ESWL, emergency URS can result in both stone disintegration and relief from colic pain. However, significant data on the ureteroscopic management of ureteral stones in an emergency setting are completely lacking. We recently published the first report focusing on ureteroscopic management of ureteral stones in emergency conditions.[42] The procedure was performed rapidly after the onset of renal colic due to ureteral stone (within 12 h from the admission to the emergency room). In our series the overall stone-free rate was 92,4%, which increased to 94,6% when only distal ureteral stones were considered. The overall complications rate was 4,2%, which decreased to 1,4% when only the smaller (less than 10 mm) stones were considered. These results resemble those from the current literature on elective URS.[43]

## CONCLUSION

The number of outpatient visits, emergency department encounters and total estimated annual expenditure for patients with claims for a diagnosis of urolithiasis have all doubled from 1994 to 2000.[44]

Recently, a number of reports have demonstrated that α-antagonists and calcium channel blockers can be used to augment spontaneous stone expulsion and improve time to expulsion of distal ureteral stones. Thus, active monitoring with the administration of MET currently represents a valid option for distal ureteral stones up to 10 mm that might have the chance for spontaneous passage. This conservative approach has also been shown to be a cost-effective strategy before embarking on surgical intervention.[45] At four to six weeks, for distal stones, irrespective of stone size, elective ESWL and URS are both acceptable treatment modalities, even if URS is mostly preferred.[3]

Emergency SWL can be easily offered for proximal symptomatic ureteral stones. In case of failure, further retreatment is possible in elective setting, either with SWL or URS.

Currently, there seems to be a shift away from noninvasive SWL in favor of more invasive ureteroscopic options. The reason for this shift is the recent advances that have been made in ureteroscopic technology, intracorporeal lithotripsy probes and extraction devices.[41,43] At the same time the trend in ESWL technology moved toward less expensive, more compact and mobile, but also less powerful machines.[46]

Although the need for rapid management of ureteral stones has been accepted, the best modality of treatment is still a matter of debate. The best procedure to choose is dependent on several factors, besides stone size and location, including operators’ experience, patient preference, available equipment and related costs.[4]

Peschel *et al.*, concluded that considerable differences between ESWL and URS can be recognized and that from the patient’s viewpoint achieving a stone-free state as soon as possible is the ultimate goal. Therefore, most patients in their study were satisfied with URS but would not have been satisfied with ESWL, mainly because of the longer time to obtain stone-free status with the latter. In our experience, the patients had their problem solved in a short period and with no need of additional bothersome auxiliary procedures, such as been reported from other series in those undergoing emergency SWL.[42]

**Figure 1:**
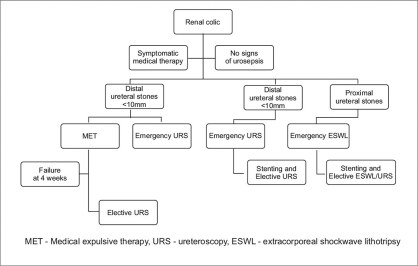
Emergency management of ureteral stones

Patient satisfaction becomes increasingly important when choosing between competing modalities of similar efficacy, and so it is difficult to give priority to either of these procedures. Operator’s experience, access to adequate equipment and specific circumstances are probably the most important determinants of which method will be most appropriate for each particular case [Figure 1].
